# COVID-19 and psychological support by phone: Demands assisted at a call center service of a brazilian university

**DOI:** 10.1192/j.eurpsy.2021.805

**Published:** 2021-08-13

**Authors:** L. Floriano, E. Pinto, D. Bordin

**Affiliations:** Nursing And Public Health, State University of Ponta Grossa, Ponta Grossa, Brazil

**Keywords:** mental health, COVID-19, Psychology

## Abstract

**Introduction:**

Throughout the COVID-19 Pandemic, it was recommended to implement mental health care services mediated by Information and Communication Technologies to alleviate the suffering caused by the social distance.

**Objectives:**

To characterize the demands and the possibilities of psychological support at the Call Center of a Brazilian public university, which aims clarifying doubts about COVID-19 and to forward suspected cases of contamination.

**Methods:**

Cross-sectional, quantitative study with secondary data. The data came from 60 Psychological Support attendance records, carried out over 24 weeks. The data were analyzed descriptively and by the paired test and McNemar.

**Results:**

The majority of consultations were for people aged among 51 and 60 years (21.7%). The main demands were social (40.0%), related to feelings (40.0%) and self-reported diagnosis (18.3%), with each service mostly split into two (31.7%) or three (31.7%) conducts. There was a significant increase in the average number of reports of symptoms of psychological distress experienced during the pandemic (p <0.001), including changes in sleep patterns (p <0.001) and appetite (p = 0.002), physical symptoms (p = 0.001), physical and emotional discomfort (p <0.001) and crying / depressive mood attacks (p = 0.002). As conducts, there was a predominance of psychoeducation (78.3%) and strategies for managing suffering (68.3%) at the expense of referrals to specialized in-person services (21.7%).

**Conclusions:**

There were several demands of the evaluated Psychological Support service, which presents an important and promising strategy for meeting the demands of psychological distress in the midst of Pandemic, especially for the adult and elderly public.

